# Molecular Characterization and Elucidation of Pathways to Identify Novel Therapeutic Targets in Pulmonary Arterial Hypertension

**DOI:** 10.3389/fphys.2021.694702

**Published:** 2021-07-23

**Authors:** Xiaoting Yao, Tian Jing, Tianxing Wang, Chenxin Gu, Xi Chen, Fengqiang Chen, Hao Feng, Huiying Zhao, Dekun Chen, Wentao Ma

**Affiliations:** College of Veterinary Medicine, Northwest A&F University, Xianyang, China

**Keywords:** systems biology, pulmonary arterial hypertension, protein-drug interaction, biomarkers, protein subcellular localization

## Abstract

**Background:** Pulmonary arterial hypertension (PAH) is a life-threatening chronic cardiopulmonary disease. However, there are limited studies reflecting the available biomarkers from separate gene expression profiles in PAH. This study explored two microarray datasets by an integrative analysis to estimate the molecular signatures in PAH.

**Methods:** Two microarray datasets (GSE53408 and GSE113439) were exploited to compare lung tissue transcriptomes of patients and controls with PAH and to estimate differentially expressed genes (DEGs). According to common DEGs of datasets, gene and protein overrepresentation analyses, protein–protein interactions (PPIs), DEG–transcription factor (TF) interactions, DEG–microRNA (miRNA) interactions, drug–target protein interactions, and protein subcellular localizations were conducted in this study.

**Results:** We obtained 38 common DEGs for these two datasets. Integration of the genome transcriptome datasets with biomolecular interactions revealed hub genes (HSP90AA1, ANGPT2, HSPD1, HSPH1, TTN, SPP1, SMC4, EEA1, and DKC1), TFs (FOXC1, FOXL1, GATA2, YY1, and SRF), and miRNAs (hsa-mir-17-5p, hsa-mir-26b-5p, hsa-mir-122-5p, hsa-mir-20a-5p, and hsa-mir-106b-5p). Protein–drug interactions indicated that two compounds, namely, nedocromil and SNX-5422, affect the identification of PAH candidate biomolecules. Moreover, the molecular signatures were mostly localized in the extracellular and nuclear areas.

**Conclusions:** In conclusion, several lung tissue-derived molecular signatures, highlighted in this study, might serve as novel evidence for elucidating the essential mechanisms of PAH. The potential drugs associated with these molecules could thus contribute to the development of diagnostic and therapeutic strategies to ameliorate PAH.

## Introduction

Pulmonary arterial hypertension (PAH) is a rare vascular disease with an annual incidence of two cases per million (Peacock et al., 2007). PAH is defined by a mean pulmonary arterial pressure (mPAP) > 20 mmHg at rest, a pulmonary artery wedge pressure (PAWP) ≤ 15 mmHg, and a pulmonary vascular resistance (PVR) ≥ 3.0 Wood units (Gouyou et al., 2021). As an obliterative vasculopathy, PAH is characterized by high pulmonary arterial pressure, resulting in right ventricular failure and even death (Boucly et al., 2017; Tang et al., 2018). Over the past decades, the progression of effective medical treatments and the application of combined therapy have significantly improved the prognosis of patients with PAH (Sitbon et al., 2014, 2016; Galie et al., 2015). Although current diagnosis and therapy strategies have effectively ameliorated the abnormal hemodynamics and severe pulmonary vascular remodeling of PAH, and efficaciously alleviated the clinical symptoms in patients with PAH, there are still a number of patients suffering from persistent symptoms and even right heart failure (Van De Veerdonk et al., 2011, 2015). Thus, investigating PAH biomarkers is essential not just for a better understanding of PAH development but also as a key step to establish promising novel treatment strategies (Kerkhof et al., 2019).

The correlation between PAH and poor cardiorespiratory outcomes justifies the necessity of early diagnosis and treatment of this disease (Farhadi et al., 2017). The wide application of cardiac catheterization and echocardiography in clinical practice, evaluation of pulmonary artery pressure and right heart hemodynamic as well as high-resolution image data are no longer difficult to access, which significantly improve the differential diagnosis and assessment of patients with PAH (Claessen and La Gerche, 2017; Farhadi et al., 2017). Indeed, there is tremendous progress in understanding the essential pathophysiology of PAH, including the prognostic biomarkers as well as many promising therapy options (Ma et al., 2020). However, there are limited studies providing PAH-associated gene expression profiles, and a number of studies implying that there may be an extraordinary significance of investigating this type of clinical molecular biomarkers and elucidating the fundamental mechanisms involved in PAH. This may be useful in developing a new scientific-based diagnosis modality and performing targeted therapy in patients with PAH more precisely (Farhadi et al., 2017; Sullivan and Kass, 2019; Ma et al., 2020; Maremanda et al., 2020).

In recent years, bioinformatics analysis has been widely employed to investigate the microarray data to estimate differentially expressed genes (DEGs) and adopt various analyses (Kanwar, 2020). However, the limited sample size or high false-positive rate of a single microarray analysis might hinder the derivation of reliable conclusions. The present study retrieved two separate microarray datasets from gene expression omnibus (GEO) for further analyses. Common DEGs between patients with PAH and controls of these two datasets were screened to identify significant biomarkers. Potential differentially expressed genes and hub genes participating in PAH were estimated *via* Gene Ontology (GO) annotations, Kyoto Encyclopedia of Genes and Genomes (KEGG) pathway enrichment analyses, protein–protein interaction (PPI) network investigations, and protein subcellular localization. Eventually, a total of 38 DEGs and 9 hub genes was chosen as prospective diagnostic candidates and targeted biomarkers for PAH.

## Materials and Methods

### Identification of the Differentially Expressed Genes of Microarray Datasets From Lung Tissue With Patients With PAH

The high-throughput datasets of microarray gene expression in PAH were retrieved from the NCBI-GEO database (Barrett et al., 2013). The datasets were obtained from two separate studies on human lung tissues that were compared across normal individuals and patients with PAH on Affymetrix microarrays, which have been deposited in the NCBI-GEO database under the accession numbers GSE53408 and GSE113439. GSE53408 contains samples of 16 individuals, including 11 controls and five patients with idiopathic pulmonary arterial hypertension (IPAH), taken from a study originally published by Zhao et al. (2014). GSE113439 encompasses 26 lung tissue samples (11 controls and 15 cases), the PAH group contains six patients with IPAH, four patients with PAH secondary to the connective tissue disease (CTD), four patients with PAH secondary to the congenital heart disease (CHD), and one patient with chronic thromboembolic pulmonary hypertension (CTEPH). This dataset was originally analyzed by Mura and colleagues to explore DEGs in PAH compared to the controls (Mura et al., 2019). To verify the results, GSE15197 (13 control and 18 PAH lung tissues) and GSE117261 (25 control and 58 PAH lung tissues) were also used in our research. First, we normalized the gene expression datasets for log_2_ transformation. Thereafter, these datasets were analyzed in GEO2R of NCBI with Limma package in hypothesis testing, and the false discovery rate was regulated by Benjamini and Hochberg correction. As the cutoff criteria, a *p* < 0.01 was considered to select the significant DEGs. Jvenn was exploited to identify the common DEGs of two datasets and make the Venn plot (Bardou et al., 2014).

### Gene Ontology and Gene Pathway Enrichment Analysis

As the bioinformatics resource, Enrichr was adopted to ascertain the common DEGs and identify their molecular functions, biological processes, cellular functions, and pathway annotations (Kuleshov et al., 2016). GO and the KEGG pathway database served as the annotation sources in this study. PANTHER database was employed to detect the over-representation of protein classes (Mi et al., 2013). Again, a *p* < 0.05 was considered significant in all enrichment analyses.

### Protein–Protein Interaction Analysis

The PPI network of proteins encoded by DEGs was constructed by the STRING protein interaction database. Since the number of common DEGs (Szklarczyk et al., 2017) was low, the PPI network was developed with a medium confidence score (600). NetworkAnalyst was performed to visualize and analyze the topological network (Xia et al., 2015). The hub proteins were selected based on the topological indices, i.e., degree > 15.

### Differentially Expressed Genes–Transcription Factor (TF) Interaction Analysis

To investigate the regulatory TFs that regulate the DEGs at the transcriptional level, we identified the interactions of TF-targeted genes with the JASPAR database and estimated the topological parameters using NetworkAnalyst (Xia et al., 2015; Khan et al., 2018).

### Differentially Expressed Genes–miRNA Interaction Analysis

The regulatory miRNAs that control DEGs at the posttranscriptional level were evaluated by identifying the interactions of miRNA-target genes with TarBase and miRTarBase and by examining the topological parameters using the NetworkAnalyst (Sethupathy et al., 2006; Hsu et al., 2011; Xia et al., 2015).

### Protein–Drug Interaction Analysis

The protein–drug interaction was estimated by the DrugBank database (version 5.1.8) *via* the NetworkAnalyst, which could highlight the potential drugs applied in the treatment of PAH (Wishart et al., 2018). The results suggested the interaction of HSP90AA1 (heat shock protein) with few drugs. Moreover, according to the protein–ligand docking server SwissDock, molecular docking analysis between HSP90AA1 and drugs was elucidated with the three-dimensional crystal structure (Grosdidier et al., 2011; Biasini et al., 2014). The protein–drug interaction analysis also indicated the interaction of HSP90AA1 with nedocromil and SNX-5422 (Wishart et al., 2018).

### Investigation of Protein Subcellular Localization

WoLF PSORT helped to determine the subcellular localization of a set of proteins encoded by DEGs (Horton et al., 2007). The subcellular localization of proteins was predicted based on their amino acid sequences. This method made predictions in the light of the known sorting signals, amino acid content, and functional motifs, collected from UniProt (Universal Protein) and GO database.

## Results

### Transcriptomic Signatures of PAH

**Figure 1:**
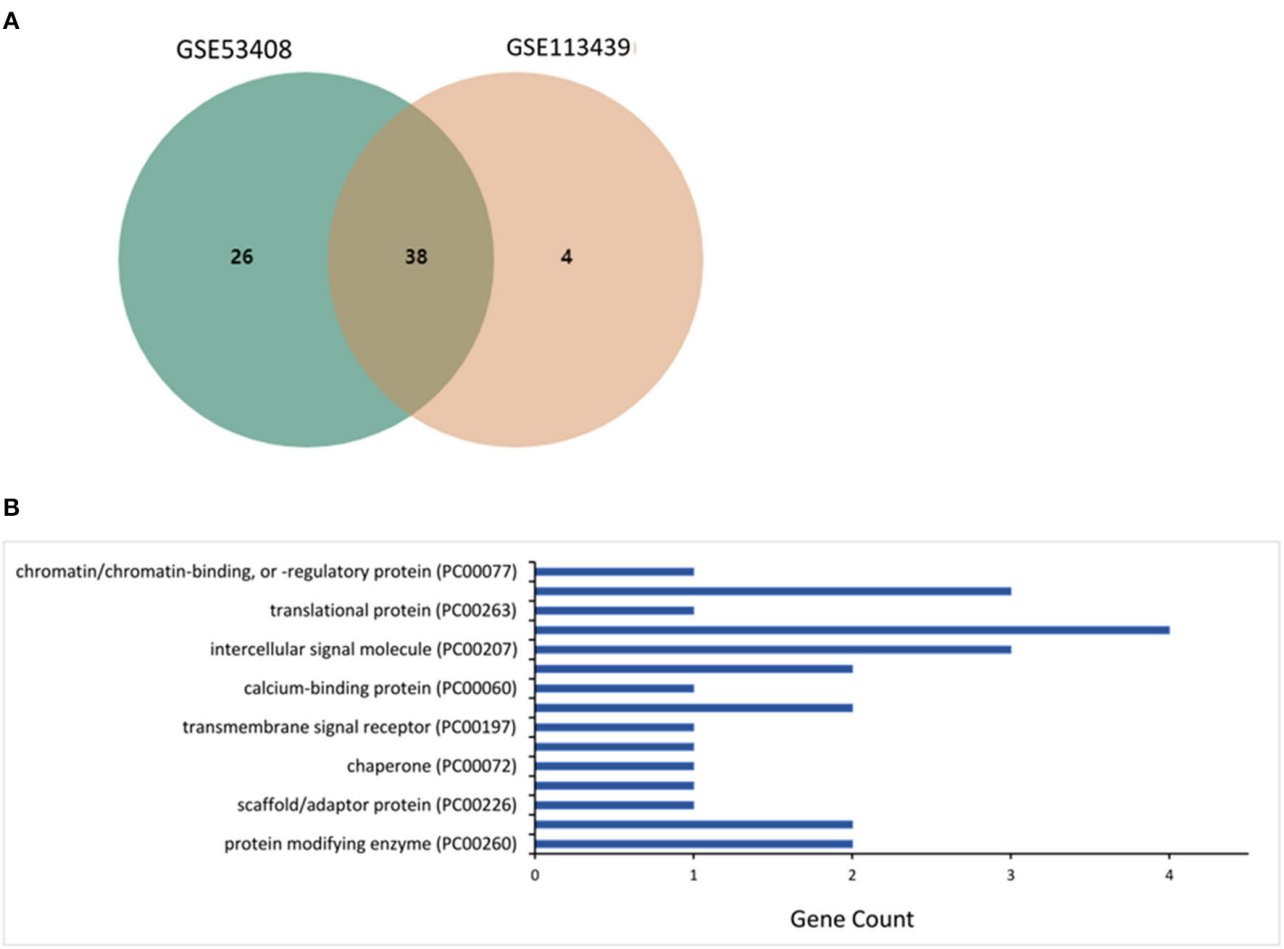
Investigation of differentially expressed genes (DEGs) in microarray datasets GSE53408 and GSE113439. **(A)** The mutual core DEGs analyzed between two datasets. **(B)** The over-represented protein class coded by the DEGs estimated using the PANTHER database.

The microarray datasets retrieved from the lung tissues of patients with PAH were investigated, and 38 mutual core DEGs (SCARNA4, GALNT1, CCDC186, EEA1, SMC4, GCC2, ZNF721, EPRS, RAD50, POSTN, DNTTIP2, RAMP2, HSP90AA1, ZNF845, HSPD1, NEXN, CFH, ZNF267, HSP90AA6P, CD163, HIGD1B, PI15, SNORD20, ANGPT2, S100A3, TDO2, SOSTDC1, ANLN, TSHZ2, SLC7A11, MS4A15, HSPH1, DKC1, TTN, VSIG1, SPP1, RNU5D-1, and MMP8) were detected between the two datasets. The core DEGs, reflecting the transcriptomic signatures for PAH (Figure 1A), were classified into eight groups based on their functions and bioactivities as protein-modifying enzyme (8%), transporter (8%), scaffold/adaptor protein (4%), membrane traffic protein (4%), chaperone (4%), cell adhesion molecule (4%), transmembrane signal receptor (4%), defense/immunity protein (8%), calcium-binding protein (4%), cytoskeletal protein (8%), intercellular signal molecule (12%), gene-specific transcriptional regulator (15%), translational protein (4%), metabolite interconversion enzyme (12%), and chromatin/chromatin-binding or chromatin-regulatory protein (4%) (Figure 1B).

Gene-set enrichment analysis revealed the abundance of DEGs in biological processes, molecular functions, and cellular components. Table 1 summarizes the specific information. The enrichment analysis of the molecular pathway claimed that the pathways involved in non-homologous end-joining, protein processing in the endoplasmic reticulum, tuberculosis, and PI3K-Akt signaling pathway were altered (Table 2).

**Table 1:** Gene set enrichment analysis for differentially expressed genes detected from the microarray data of lung tissue with patients with pulmonary arterial hypertension (PAH).

**Category**	**GO ID**	**Term**	***P*-value**	**Genes**
Biological process	GO:0048739	Cardiac muscle fiber development	0.0002	NEXN; TTN
	GO:0007076	Mitotic chromosome condensation	0.0004	SMC4; TTN
	GO:0051131	Chaperone-mediated protein complex assembly	0.0005	HSP90AA1; HSPD1
	GO:0035886	Vascular smooth muscle cell differentiation	0.0113	RAMP2
	GO:0014897	Striated muscle hypertrophy	0.0113	TTN
	GO:0072012	Glomerulus vasculature development	0.0113	ANGPT2
	GO:0051133	Regulation of NK T-cell activation	0.0113	HSPH1
	GO:0097647	Amylin receptor signaling pathway	0.0113	RAMP2
	GO:0045343	Regulation of MHC class I biosynthetic process	0.0113	HSPH1
	GO:0097084	Vascular smooth muscle cell development	0.0113	RAMP2
Molecular function	GO:0098821	BMP receptor activity	0.0113	SOSTDC1
	GO:0004017	Adenylate kinase activity	0.0132	RAD50
	GO:0051880	G-quadruplex DNA binding	0.0132	RAD50
	GO:0031433	Telethonin binding	0.0132	TTN
	GO:0000014	Single-stranded DNA endodeoxyribonuclease activity	0.0132	RAD50
	GO:0036122	BMP binding	0.0170	SOSTDC1
	GO:0003691	Double-stranded telomeric DNA binding	0.0170	RAD50
	GO:0000774	Adenyl-nucleotide exchange factor activity	0.0207	HSPH1
	GO:0030554	Adenyl nucleotide binding	0.0207	HSPH1
	GO:0043047	Single-stranded telomeric DNA binding	0.0226	RAD50
Cellular component	GO:0071682	Endocytic vesicle lumen	0.0005	HSP90AA1; HSPH1
	GO:0019907	Cyclin-dependent protein kinase activating kinase holoenzyme complex	0.0132	HSPD1
	GO:0038037	G-protein-coupled receptor dimeric complex	0.0151	RAMP2
	GO:0000794	Condensed nuclear chromosome	0.0019	RAD50; TTN
	GO:0099738	Cell cortex region	0.0207	ANLN
	GO:0000793	Condensed chromosome	0.0026	RAD50; TTN
	GO:0019908	Nuclear cyclin-dependent protein kinase holoenzyme complex	0.0300	HSPD1
	GO:0000228	Nuclear chromosome	0.0044	RAD50; TTN
	GO:0005865	Striated muscle thin filament	0.0355	TTN
	GO:0030139	Endocytic vesicle	0.0179	HSP90AA1; HSPH1

*The top 10 abundant Gene Ontology (GO) terms were tabulated*.

**Table 2:** The enriched molecular pathways of differentially expressed genes (DEGS) in PAH disease.

**Pathways**	***P*-value**	**Associated genes**
Non-homologous end-joining	0.0244	RAD50
Protein processing in endoplasmic reticulum	0.0392	HSP90AA1; HSPH1
Tuberculosis	0.0454	EEA1; HSPD1
PI3K-Akt signaling pathway	0.0294	HSP90AA1; ANGPT2; SPP1

### Proteomic Signatures in PAH

**Figure 2:**
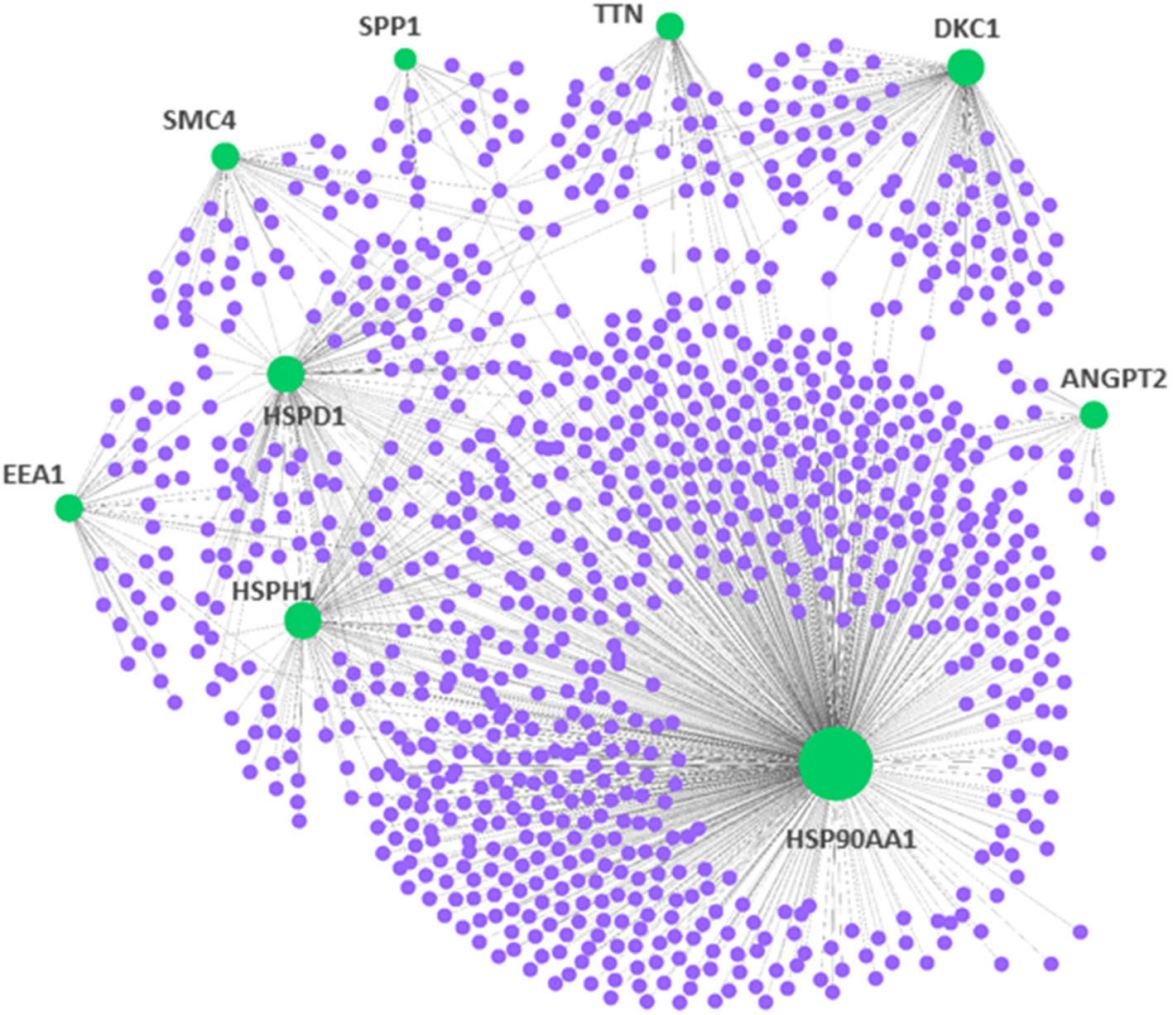
Protein–protein interaction network for the DEGs in pulmonary arterial hypertension (PAH). The nodes indicated the DEGs, while the edges indicated the interactions between different proteins. The medium confidence score was performed to construct the Protein–protein interaction (PPI) networks.

To demonstrate central proteins, the protein–protein network of mutual DEGs was constructed to offer deep knowledge in the biological characterization of targeted proteins encoded by DEGs and the estimation of drug targets (Figure 2). The hub proteins responsible for transmitting signal stimulus to other proteins in networks are obtained from the topological examination of PPI networks. Based on the topological metric, the present method estimated the hub proteins, which could serve as biomarkers and drug targets in PAH. Besides, Table 3 highlights nine central hub proteins (HSP90AA1, ANGPT2, HSPD1, HSPH1, TTN, SPP1, SMC4, EEA1, and DKC1). The other two independent studies also showed that these hub proteins play an important role in PAH (Supplementary Table 1).

**Table 3:** Summary of hub proteins identified from protein–protein interactions analysis of encoded differentially expressed genes in PAH disease.

**Symbol**	**Description**	**Feature**
HSP90AA1	Heat shock protein	Influenced the progression of pulmonary disease (Deng et al., 2021).
ANGPT2	Protein marker and mediator	Participated in the direct regulation of inflammation-related signal pathways in PAH (Zhong et al., 2018).
HSPD1	Heat shock protein	Involved in pulmonary disease as differential expression gene (Maremanda et al., 2020).
HSPH1	Heat shock protein	Interacted with STAT3 and enhanced its phosphorylation in acute lung injury (Liang et al., 2020).
TTN	TITIN protein	Served as a pathogenic gene associated with total anomalous pulmonary venous connection (Shi et al., 2018).
SPP1	Secreted phosphoprotein	Activated the idiopathic pulmonary fibrosis myofibroblasts in lung fibrosis (Morse et al., 2019).
SMC4	A core subunit of condensin complexes	Enriched in facilitating mitotic cell cycle process in PAH (Luo et al., 2020).
EEA1	Early endosome antigen-1	Contained sorting endosomes which marked early endosomes (Chrifi et al., 2019).
DKC1	Dyskeratosis congenita 1	Encoded the protein dyskerin and maintained telomeres in pulmonary disease (Khincha et al., 2014).

### Regulatory Signatures of PAH

**Figure 3:**
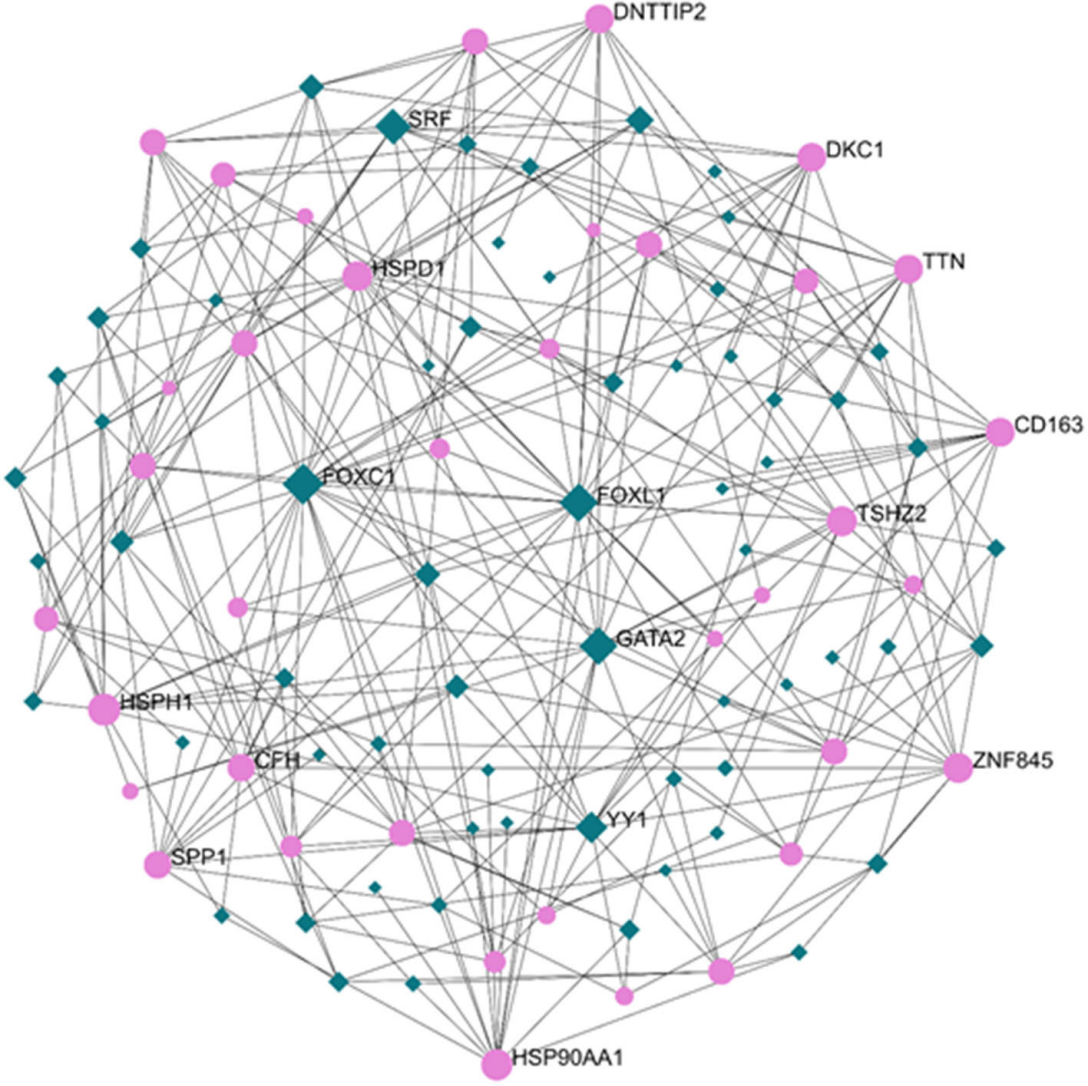
Regulatory networks showed the DEG–transcription factor (DEG–TF) interactions in PAH. Medium confidence score was performed to construct the regulatory networks. Green, TFs; pink, DEGs.

**Figure 4:**
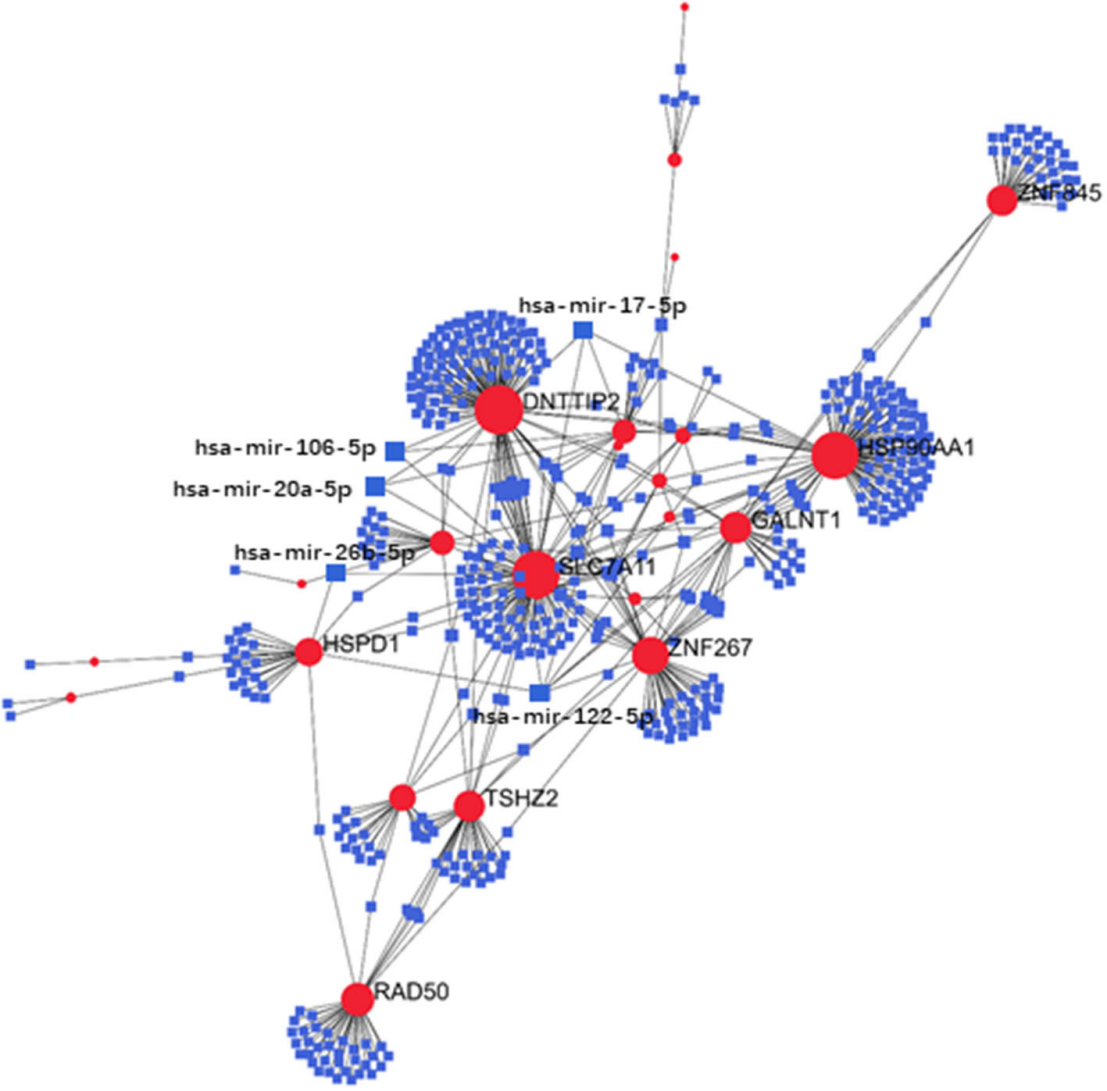
Regulatory networks showed the DEG–miRNA interactions in PAH. Medium confidence score was performed to construct the regulatory networks. Blue, miRNAs; red, DEGs.

Exploration of the DEG–TF interaction (Figure 3) and DEG–miRNA interaction (Figure 4) with topological parameters contributed to the identification of the central regulatory biomolecules of PAH. As detailed in Table 4, the present study documented five TFs (FOXC1, FOXL1, GATA2, YY1, and SRF) and five miRNAs (hsa-mir-17-5p, hsa-mir-26b-5p, hsa-mir-122-5p, hsa-mir-20a-5p, and hsa-mir-106b-5p).

**Table 4:** Summary of the regulatory biomolecules [transcription factors (TFs), miRNAs] of DEGs in PAH disease identified from the interactions of DEGs with TFs and DEGs with miRNAs.

**Symbol**	**Description**	**Feature**
**TFs**		
FOXC1	Forkhead box C1	Afflicted with PAH (Yang et al., 2020).
FOXL1	Forkhead box L1	Afflicted with PAH (Stankiewicz et al., 2009).
GATA2	GATA binding protein 2	Afflicted with PAH (Jouneau et al., 2017).
YY1	YY1 transcription factor	Afflicted with PAH (Zhang L. et al., 2021).
SRF	Serum response factor	Afflicted with PAH (Ding et al., 2017).
**miRNAs**		
hsa-mir-17-5p	MicroRNA 17	Hypoxia-induced pulmonary vascular smooth muscle cell proliferation in PAH (Liu et al., 2018).
hsa-mir-26b-5p	MicroRNA 26	Afflicted with PAH (Chouvarine et al., 2020).
hsa-mir-122-5p	MicroRNA 122	Have a good diagnostic performance in PAH (Zhang et al., 2018).
hsa-mir-20a-5p	MicroRNA 20	Promoted pulmonary artery smooth muscle cell proliferation in PAH (Zhou et al., 2020).
hsa-mir-106b-5p	MicroRNA 106	Suppress the migration of pulmonary artery smooth muscle cell in PAH (Chen et al., 2020).

### Protein–Drug Interactions

**Figure 5:**
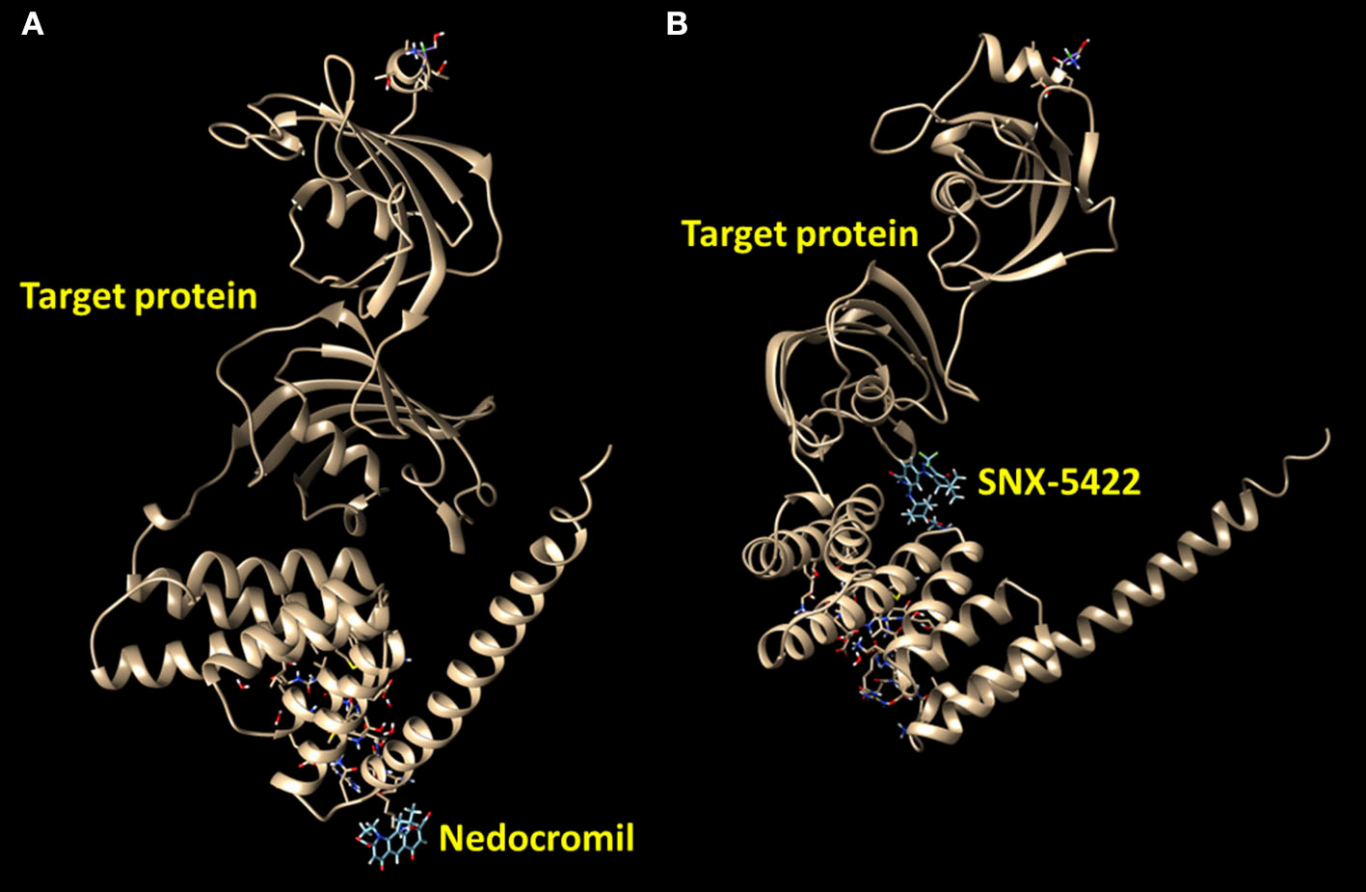
Predict binding modes of protein–drug interactions. Molecular graphics images were produced by the UCSF Chimera package. Binding modes were scored using their FullFitness and clustered. Clusters were then ranked based on the average FullFitness of their elements. **(A)** Nedocromil–protein; **(B)** SNX-5422–protein.

The protein–drug interaction network reported the relation of HSP90AA1 protein with the drugs nedocromil and SNX-5422 (Figure 5, Table 5). Nedocromil is a pyranoquinolone derivative that can suppress the activation of inflammatory cells, such as eosinophils, neutrophils, macrophages, mast cells, monocytes, and platelets. SNX-5422 is not only an oral agent exhibiting strong efficacy and tolerability but also a new synthetic Hsp90 inhibitor, and it was considered as a drug with breakthrough treatment and widespread applicability in a wide range of cancers. According to the statistical significance threshold of protein–drug interaction and the potential influence of the targeted protein in PAH pathogenesis, several protein–drug interactions were screened, and molecular docking simulations were carried out to determine the binding affinities of the drugs with targeted proteins (Table 5). The resultant energetic states and docking scores confirmed the thermodynamic feasibility of all these interactions.

**Table 5:** Protein–drug interactions and their binding affinity by molecular docking statistics.

**Target protein**	**Drug**	**Description**	**Full fitness (kcal/mol)**	**Estimated ΔG (kcal/mol)**
HSP90AA1	Nedocromil	A pyranoquinolone derivative	−2869.51	−8.52
	SNX-5422	A synthetic, novel, small-molecule Hsp90 inhibitor	−2815.99	−7.49

### Protein Subcellular Localization

The WoLF PSORT software package can predict the subcellular localization of proteins encoded by the 38 DEGs in PAH. The proportions of these proteins in distinct subcellular compartments were computed, which indicated the localization of 89.5% of the proteins in extracellular areas, whereas the remaining 10.5% existed in the nuclear area. Notably, all hub proteins manifested the extracellular localization, including HSP90AA1, ANGPT2, HSPD1, HSPH1, TTN, SPP1, SMC4, EEA1, and DKC1.

## Discussion

The diagnosis of PAH currently relies on the right heart catheterization, but the specific biomarkers for PAH diagnosis are an unmet challenge. The present research focused on the comprehensive analysis of the gene expression patterns in lung tissues of patients with PAH and exploration of the robust candidate molecular targets, which may act as potential biomarkers of PAH. Our study may, therefore, provide the relevant information regarding the progression of PAH.

With a wide application in biomedical investigation, microarray datasets have become a major resource for elucidating biomarker candidates (Budinska et al., 2013; Marisa et al., 2013). The extensive contribution of microarray gene expression profiling has also been reflected in the investigation of DEGs in various diseases, including Alzheimer's disease and breast cancer (Nami and Wang, 2018; Rahman et al., 2020). Significant alterations in the profiles of 38 genes in two separate transcriptomic datasets have been observed from the gene expression patterns in the lung tissues of patients with PAH. The enrichment analysis reported PAH-related molecular pathways in the endoplasmic reticulum and PI3K-Akt signaling pathway (Table 2) (Li et al., 2016).

Interactive analysis of PPI networks facilitates the identification of the proteins that play a key role in the pathophysiology of various diseases (Goh et al., 2007; Yang et al., 2009). The PPI analysis offers deep knowledge in the biological characterization of targeted proteins encoded by DEGs and the estimation of drug targets (Taz et al., 2021; Xu et al., 2021; Zhang T. et al., 2021). In light of DEGs, PPI networks detected a set of key hub proteins (Table 3), which may reflect the onset and development processes of vascular diseases. These hub proteins were involved in a number of biological and pathologic processes. As a hub protein, HSP90AA1 was differentially expressed in the lung tissues of patients with PAH and indulged in the inflammatory responses of the airway and the smooth muscle function of the bronchia, which reflected that HSP90AA1 may be a potential prognostic biomarker and drug target for the treatment of PAH (Deng et al., 2021). Consistent with our findings, ANGPT2 was upregulated in several inflammatory diseases and took part in the direct regulation of inflammation-related signal pathways in PAH (Zhong et al., 2018). Differential expression of HSPD1, involved in mitochondrial biogenesis, was noted in patients suffering from pulmonary diseases compared with normal controls (Maremanda et al., 2020). Interaction of HSPH1 with STAT3 may enhance its phosphorylation, which exacerbated pulmonary inflammation in acute lung injury, though there is no such report relating to PAH (Liang et al., 2020). Furthermore, TTN, a pathogenic gene in total anomalous pulmonary venous connection, appeared to play a critical role in the genetic mechanism (Shi et al., 2018). It has been suggested that TTN isoform composition was unchanged in PAH cardiomyocytes but that TTN phosphorylation was significantly decreased in patients with PAH (Rain et al., 2013). Pulmonary myofibroblasts can be activated by highly proliferative SPP1 macrophages, in turn, contributing vitally to lung fibrosis (Morse et al., 2019). Previous studies have shown that the SPP1 gene was significantly increased in PAH and played an important role by promoting pulmonary vascular smooth muscle cell (PVSMC) proliferation (Saker et al., 2016; Zeng et al., 2021). SMC4 manifested higher expression levels in patients with PAH (Luo et al., 2020). Similarly, SMC4 is a vital core subunit of condensin, which has an essential impact on mitotic chromosome condensation (Takemoto et al., 2004; Luo et al., 2020). It has been suggested that knockdown of SMC4 could inhibit Toll-like receptor-mediated production of several proinflammatory cytokines, including IL-6 and TNF-α in macrophages (Ma et al., 2020). Interaction of EEA1 with platelet-derived growth factor receptor can mark early endosomes, which are required for the progression of secondary pulmonary alveolar septa, which reflected that the internalization of TGF-R by clathrin-mediated endocytosis in EEA1-positive endosomes resulted in productive nuclear signaling *via* the interaction of TGF-R with Smad anchor for receptor activation (SARA) (Saker et al., 2016; Chrifi et al., 2019). DKC1 was known as a gene-encoded protein dyskerin and affected several modules of the telomere complex in pulmonary disease (Heiss et al., 1998; Khincha et al., 2014).

The interaction of DEGs with TFs and DEGs with miRNAs was also examined to highlight the transcriptional or posttranscriptional regulators associated with the mutual DEGs. Table 4 illustrates a set of regulators related to DEGs in PAH, including TFs and miRNAs. The measured transcriptional regulatory TFs (FOXC1, FOXL1, GATA2, YY1, SRF) that interacted with DEGs in PAH were in accordance with the previous observations (Stankiewicz et al., 2009; Ding et al., 2017; Yang et al., 2019, 2020; Zhang L. et al., 2021). miRNA is known as a single-stranded non-coding RNA that targets their transcripts to regulate gene expressions (Caruso et al., 2017). As potential biomarkers, miRNAs may provide breakthrough treatment strategies for the diagnosis and management of PAH (Caruso et al., 2017). Therefore, the miRNAs were explored as regulatory factors of target DEGs. The mir-17-5p identified in this study was involved in the proliferation of pulmonary vascular smooth muscle cells and could thus provide a potential novel treatment target for the control of PAH (Liu et al., 2018). In comparison with the control group, the lower concentration of miR-26b-5p in the lung tissue of patients with PAH signified its involvement in the remodeling process of PAH (Chouvarine et al., 2020). As a good biomarker for hypertension, mir-122-5p had a prominent diagnostic performance, and its dysregulation could indulge in the risk of PAH (Zhang et al., 2018). The mir-20a-5p-induced proliferation of pulmonary artery smooth muscle cells can exacerbate the development of PAH *via* targeting ATP-binding cassette subfamily A1 (Zhou et al., 2020). The mir-106b-5p played a key role in suppressing the migration of pulmonary artery smooth muscle cells, indicating that mir-106b-5p may act as a potential marker in PAH (Chen et al., 2020). These biomolecules may regulate target genes at transcriptional or posttranscriptional levels.

Given the importance of hub genes and their potential role in the pathogenic process in PAH, the interactions between target proteins and drugs were further studied in this research. Two drugs were spotted from the interaction network according to the DrugBank database (Table 5). A previous study has suggested that nedocromil sodium could inhibit antigen-induced shrinkage of human lung parenchymal as well as bronchial strips (Napier et al., 1990). As a therapy for non-small-cell lung cancer (NSCLC), another study also supported the evaluation of SNX-5422, especially in cases where cancer was driven by c-Met amplification and mutated epidermal growth factor receptor (EGFR) forms that were resistant to EGFR inhibitors (Rice et al., 2009). Several molecular modeling-based techniques were conducted in pharmaceutical research to evaluate complex biological systems, especially for molecular docking methods, which were broadly applied in drug designs to demonstrate the ligand conformation with binding sites of target proteins. The free energy was estimated in molecular docking methods by evaluating critical phenomena that participated in the intermolecular recognition processes in ligand–receptor binding (Chen et al., 2020). Henceforth, the binding modes of drugs with target protein HSP90AA1 were screened, and the energetically stable conformations were obtained from the existing databases (Figure 5). Moreover, the protein subcellular localizations for DEGs confirmed that extracellular and nuclear areas were the primary subcellular sites for the DEGs. This result substantiated the participation of these genes in PAH, extending from the nucleus to extracellular areas. For further drug selection to alleviate PAH, the protein subcellular localization may provide targeting sites for specific drugs. The present study documented the relationship between drugs and putative PAH molecular biomarkers; however, the consequence of molecular targets blockade was ambiguous from this study that should be taken into account for further investigation. Although the molecules are lung tissue-based and how they participate in pulmonary pathogenesis is still not known, clinical evidence has substantiated the effect of several of these identified drugs on PAH, and it may be helpful to know what are their influences on lung tissues.

## Conclusion

Integrative multi-omics analysis was adopted for the evaluation of the lung tissue-based transcriptomic profiles to identify the molecular signatures at protein levels (hub proteins, TFs) and RNA levels (mRNAs, miRNAs). Based on the genome transcriptome datasets, nine hub genes (HSP90AA1, ANGPT2, HSPD1, HSPH1, TTN, SPP1, SMC4, EEA1, and DKC1) were found in this study. The significant abundance of numerous pivotal hub genes was evident in pathways that participated in endoplasmic reticulum-related protein processing and PI3K-Akt signaling pathway. We also screened TFs (FOXC1, FOXL1, GATA2, YY1, and SRF) and miRNAs (hsa-mir-17-5p, hsa-mir-26b-5p, hsa-mir-122-5p, hsa-mir-20a-5p, and hsa-mir-106b-5p) regulating the expression of DEGs in PAH. These biomolecules could be accounted as candidate system biomarkers at protein levels and RNA levels. Therefore, the potential molecular signatures obtained for PAH could be detected as transcripts in lung tissues, and these signatures warrant clinical analyses in patients with PAH to access their utility. The availability of these biomolecules in pulmonary tissues presumes the establishment of these biomarkers as a novel aspect of PAH development and progression.

## Data Availability Statement

The original contributions generated for the study are included in the article/Supplementary Material, further inquiries can be directed to the corresponding author/s.

## Author Contributions

DC, HZ, and WM conceived and designed the experiments. XY and TJ performed all experiments. XY, TW, and XC collected and analyzed the data. CG, HF, TJ, and FC drafted the manuscript. All authors have read and agreed to the published version of the manuscript.

## Conflict of Interest

The authors declare that the research was conducted in the absence of any commercial or financial relationships that could be construed as a potential conflict of interest.

## Publisher's Note

All claims expressed in this article are solely those of the authors and do not necessarily represent those of their affiliated organizations, or those of the publisher, the editors and the reviewers. Any product that may be evaluated in this article, or claim that may be made by its manufacturer, is not guaranteed or endorsed by the publisher.

## References

[B1] Bardou P. Mariette J. Escudie F. Djemiel C. Klopp C. (2014). jvenn: an interactive Venn diagram viewer. BMC Bioinform. 15:293. 10.1186/1471-2105-15-293 25176396 PMC4261873

[B2] Barrett T. Wilhite S. E. Ledoux P. Evangelista C. Kim I. F. Tomashevsky M.. (2013). NCBI GEO: archive for functional genomics data sets-update. Nucleic Acids Res. 41, D991–D995. 10.1093/nar/gks1193 23193258 PMC3531084

[B3] Biasini M. Bienert S. Waterhouse A. Arnold K. Studer G. Schmidt T.. (2014). SWISS-MODEL: modelling protein tertiary and quaternary structure using evolutionary information. Nucleic Acids Res. 42, W252–W258. 10.1093/nar/gku340 24782522 PMC4086089

[B4] Boucly A. Weatherald J. Savale L. Jais X. Cottin V. Prevot G.. (2017). Risk assessment, prognosis and guideline implementation in pulmonary arterial hypertension. Europ. Resp. J. 50:2017. 10.1183/13993003.00889-2017 28775050

[B5] Budinska E. Popovici V. Tejpar S. D'ario G. Lapique N. Sikora K. O.. (2013). Gene expression patterns unveil a new level of molecular heterogeneity in colorectal cancer. J. Pathol. 231, 63–76. 10.1002/path.4212 23836465 PMC3840702

[B6] Caruso P. Dunmore B. J. Schlosser K. Schoors S. Dos Santos C. Perez-Iratxeta C.. (2017). Identification of MicroRNA-124 as a major regulator of enhanced endothelial cell glycolysis in pulmonary arterial hypertension via PTBP1 (Polypyrimidine Tract Binding Protein) and pyruvate kinase M2. Circulation 136:2451. 10.1161/Circulationaha.117.028034 28971999 PMC5736425

[B7] Chen H. Ma Q. Zhang J. Meng Y. Pan L. Tian H. (2020). miR106b5p modulates acute pulmonary embolism via NOR1 in pulmonary artery smooth muscle cells. Int. J. Mol. Med. 45, 1525–1533. 10.3892/ijmm.2020.4532 32323756 PMC7138273

[B8] Chouvarine P. Geldner J. Giagnorio R. Legchenko E. Bertram H. Hansmann G. (2020). Trans-right-ventricle and transpulmonary MicroRNA gradients in human pulmonary arterial hypertension. Pediatr. Crit. Care Med. 21, 340–349. 10.1097/PCC.0000000000002207 31876555

[B9] Chrifi I. Louzao-Martinez L. Brandt M. M. Van Dijk C. G. M. Burgisser P. E. Zhu C.. (2019). CMTM4 regulates angiogenesis by promoting cell surface recycling of VE-cadherin to endothelial adherens junctions. Angiogenesi. 22, 75–93. 10.1007/s10456-018-9638-1 30097810 PMC6510885

[B10] Claessen G. La Gerche A. (2017). Pulmonary vascular function during exercise: progressing toward routine clinical use. Circ. Cardiovasc. Imaging 10:326. 10.1161/CIRCIMAGING.117.006326 28360265

[B11] Deng Y. X. Zhong J. Liu Z. J. Wang X. Q. Zhang B. (2021). Active ingredients targeting Nrf2 in the Mongolian medicine Qiwei Putao powder: systematic pharmacological prediction and validation for chronic obstructive pulmonary disease treatment. J. Ethnopharm. 265:113385. 10.1016/j.jep.2020.113385 32920133

[B12] Ding X. Zhou S. Li M. Cao C. Wu P. Sun L.. (2017). Upregulation of SRF is associated with hypoxic pulmonary hypertension by promoting viability of smooth muscle cells via increasing expression of Bcl-2. J. Cell Biochem. 118, 2731–2738. 10.1002/jcb.25922 28176371

[B13] Farhadi R. Rafiei A. Hamdamian S. Zamani H. Yazdani J. (2017). Pentraxin 3 in neonates with and without diagnosis of pulmonary hypertension. Clin. Biochem. 50, 223–227. 10.1016/j.clinbiochem.2016.11.009 27838407

[B14] Galie N. Barbera J. A. Frost A. E. Ghofrani H. A. Hoeper M. M. Mclaughlin V. V.. (2015). Initial use of ambrisentan plus tadalafil in pulmonary arterial hypertension. N. Engl. J. Med. 373, 834–844. 10.1056/NEJMoa1413687 26308684

[B15] Goh K. I. Cusick M. E. Valle D. Childs B. Vidal M. Barabasi A. L. (2007). The human disease network. Proc. Natl. Acad. Sci. U.S.A. 104, 8685–8690. 10.1073/pnas.0701361104 17502601 PMC1885563

[B16] Gouyou B. Grun K. Kerschenmeyer A. Villa A. Matasci M. Schrepper A.. (2021). Therapeutic evaluation of antibody-based targeted delivery of interleukin 9 in experimental pulmonary hypertension. Int. J. Mol. Sci. 22:460. 10.3390/ijms22073460 33801620 PMC8037792

[B17] Grosdidier A. Zoete V. Michielin O. (2011). SwissDock, a protein-small molecule docking web service based on EADock DSS. Nucleic Acids Res. 39, W270–277. 10.1093/nar/gkr366 21624888 PMC3125772

[B18] Heiss N. S. Knight S. W. Vulliamy T. J. Klauck S. M. Wiemann S. Mason P. J.. (1998). X-linked dyskeratosis congenita is caused by mutations in a highly conserved gene with putative nucleolar functions. Nat. Genet. 19, 32–38. 10.1038/ng0598-32 9590285

[B19] Horton P. Park K. J. Obayashi T. Fujita N. Harada H. Adams-Collier C. J.. (2007). WoLF PSORT: protein localization predictor. Nucleic Acids Res. 35, W585–587. 10.1093/nar/gkm259 17517783 PMC1933216

[B20] Hsu S. D. Lin F. M. Wu W. Y. Liang C. Huang W. C. Chan W. L.. (2011). miRTarBase: a database curates experimentally validated microRNA-target interactions. Nucleic Acids Res. 39, D163–169. 10.1093/nar/gkq1107 21071411 PMC3013699

[B21] Jouneau S. Ballerie A. Kerjouan M. Demant X. Blanchard E. Lederlin M. (2017). Haemodynamically proven pulmonary hypertension in a patient with GATA2 deficiency-associated pulmonary alveolar proteinosis and fibrosis. Eur. Respir. J. 49:2017. 10.1183/13993003.00407-2017 28495697

[B22] Kanwar M. K. (2020). Biomarkers in pulmonary arterial hypertension: moving closer toward precision medicine? J. Heart Lung Transplant. 39, 287–288. 10.1016/j.healun.2020.02.020 32199588

[B23] Kerkhof P. L. M. Li J. K. Handly N. (2019). Interpretation of a new biomarker for the right ventricle introduced to evaluate the severity of pulmonary arterial hypertension. Pulm. Circ. 9:2045894019826945. 10.1177/2045894019826945 30638434 PMC6540503

[B24] Khan A. Fornes O. Stigliani A. Gheorghe M. Castro-Mondragon J. A. Van Der Lee R.. (2018). JASPAR 2018: update of the open-access database of transcription factor binding profiles and its web framework. Nucleic Acids Res. 46, D260–D266. 10.1093/nar/gkx1126 29140473 PMC5753243

[B25] Khincha P. P. Wentzensen I. M. Giri N. Alter B. P. Savage S. A. (2014). Response to androgen therapy in patients with dyskeratosis congenita. Br. J. Haematol. 165, 349–357. 10.1111/bjh.12748 24666134 PMC3984599

[B26] Kuleshov M. V. Jones M. R. Rouillard A. D. Fernandez N. F. Duan Q. Wang Z.. (2016). Enrichr: a comprehensive gene set enrichment analysis web server 2016 update. Nucleic Acids Res. 44, W90–97. 10.1093/nar/gkw377 27141961 PMC4987924

[B27] Li A. Zhang S. Li J. Liu K. Huang F. Liu B. (2016). Metformin and resveratrol inhibit Drp1-mediated mitochondrial fission and prevent ER stress-associated NLRP3 inflammasome activation in the adipose tissue of diabetic mice. Mol. Cell Endocrinol. 434, 36–47. 10.1016/j.mce.2016.06.008 27276511

[B28] Liang Y. Luo J. Yang N. Wang S. Ye M. Pan G. (2020). Activation of the IL-1beta/KLF2/HSPH1 pathway promotes STAT3 phosphorylation in alveolar macrophages during LPS-induced acute lung injury. Biosci. Rep. 40:72. 10.1042/BSR20193572 32091104 PMC7056450

[B29] Liu G. Hao P. Xu J. Wang L. Wang Y. Han R.. (2018). Upregulation of microRNA-17-5p contributes to hypoxia-induced proliferation in human pulmonary artery smooth muscle cells through modulation of p21 and PTEN. Respir Res. 19, 200. 10.1186/s12931-018-0902-0 30305109 PMC6180506

[B30] Luo J. Li H. Liu Z. Li C. Wang R. Fang J.. (2020). Integrative analyses of gene expression profile reveal potential crucial roles of mitotic cell cycle and microtubule cytoskeleton in pulmonary artery hypertension. BMC Med Genomics 13:86. 10.1186/s12920-020-00740-x 32586319 PMC7318763

[B31] Ma Y. Chen S. S. Feng Y. Y. Wang H. L. (2020). Identification of novel biomarkers involved in pulmonary arterial hypertension based on multiple-microarray analysis. Biosci. Rep. 40:2346. 10.1042/BSR20202346 32886110 PMC7494994

[B32] Maremanda K. P. Sundar I. K. Li D. Rahman I. (2020). Age-dependent assessment of genes involved in cellular senescence, telomere and mitochondrial pathways in human lung tissue of smokers, COPD and IPF: associations with SARS-CoV-2 COVID-19 ACE2-TMPRSS2-Furin-DPP4 axis. medRxiv. 10.1101/2020.06.14.20129957 33013423 PMC7510459

[B33] Marisa L. De Reynies A. Duval A. Selves J. Gaub M. P. Vescovo L.. (2013). Gene expression classification of colon cancer into molecular subtypes: characterization, validation, and prognostic value. PLoS Med. 10:e1001453. 10.1371/journal.pmed.1001453 23700391 PMC3660251

[B34] Mi H. Muruganujan A. Thomas P. D. (2013). PANTHER in 2013: modeling the evolution of gene function, and other gene attributes, in the context of phylogenetic trees. Nucleic Acids Res. 41(Database issue), D377–386. 10.1093/nar/gks1118 23193289 PMC3531194

[B35] Morse C. Tabib T. Sembrat J. Buschur K. L. Bittar H. T. Valenzi E.. (2019). Proliferating SPP1/MERTK-expressing macrophages in idiopathic pulmonary fibrosis. Eur. Respir. J. 54:2018. 10.1183/13993003.02441-2018 31221805 PMC8025672

[B36] Mura M. Cecchini M. J. Joseph M. Granton J. T. (2019). Osteopontin lung gene expression is a marker of disease severity in pulmonary arterial hypertension. Respirology 24, 1104–1110. 10.1111/resp.13557 30963672

[B37] Nami B. Wang Z. (2018). Genetics and Expression Profile of the Tubulin Gene Superfamily in Breast Cancer Subtypes and Its Relation to Taxane Resistance. Cancers 10:274. 10.3390/cancers10080274 30126203 PMC6116153

[B38] Napier F. E. Shearer M. A. Temple D. M. (1990). Nedocromil sodium inhibits antigen-induced contraction of human lung parenchymal and bronchial strips, and the release of sulphidopeptide-leukotriene and histamine from human lung fragments. Br. J. Pharmacol. 100, 247–250. 10.1111/j.1476-5381.1990.tb15790.x 1696152 PMC1917434

[B39] Peacock A. J. Murphy N. F. Mcmurray J. J. Caballero L. Stewart S. (2007). An epidemiological study of pulmonary arterial hypertension. Eur. Respir. J. 30, 104–109. 10.1183/09031936.00092306 17360728

[B40] Rahman M. R. Islam T. Zaman T. Shahjaman M. Karim M. R. Huq F.. (2020). Identification of molecular signatures and pathways to identify novel therapeutic targets in Alzheimer's disease: insights from a systems biomedicine perspective. Genomics 112, 1290–1299. 10.1016/j.ygeno.2019.07.018 31377428

[B41] Rain S. Handoko M. L. Trip P. Gan C. T. Westerhof N. Stienen G. J.. (2013). Right ventricular diastolic impairment in patients with pulmonary arterial hypertension. Circulation 128, 2016–2025, 2011–2010. 10.1161/CIRCULATIONAHA.113.001873 24056688

[B42] Rice J. W. Veal J. M. Barabasz A. Foley B. Fadden P. Scott A.. (2009). Targeting of multiple signaling pathways by the Hsp90 inhibitor SNX-2112 in EGFR resistance models as a single agent or in combination with erlotinib. Oncol Res. 18, 229–242. 10.3727/096504009x12596189659240 20225761

[B43] Saker M. Lipskaia L. Marcos E. Abid S. Parpaleix A. Houssaini A.. (2016). Osteopontin, a key mediator expressed by senescent pulmonary vascular cells in pulmonary hypertension. Arterioscler. Thromb. Vasc. Biol. 36, 1879–1890. 10.1161/ATVBAHA.116.307839 27444202

[B44] Sethupathy P. Corda B. Hatzigeorgiou A. G. (2006). TarBase: a comprehensive database of experimentally supported animal microRNA targets. RNA. 12, 192–197. 10.1261/rna.2239606 16373484 PMC1370898

[B45] Shi X. Cheng L. Jiao X. Chen B. Li Z. Liang Y.. (2018). Rare copy number variants identify novel genes in sporadic total anomalous pulmonary vein connection. Front. Genet. 9:559. 10.3389/fgene.2018.00559 30532766 PMC6265481

[B46] Sitbon O. Jais X. Savale L. Cottin V. Bergot E. Macari E. A.. (2014). Upfront triple combination therapy in pulmonary arterial hypertension: a pilot study. Eur. Respir. J. 43, 1691–1697. 10.1183/09031936.00116313 24627535

[B47] Sitbon O. Sattler C. Bertoletti L. Savale L. Cottin V. Jais X.. (2016). Initial dual oral combination therapy in pulmonary arterial hypertension. Eur Respir J. 47, 1727–1736. 10.1183/13993003.02043-2015 26989105

[B48] Stankiewicz P. Sen P. Bhatt S. S. Storer M. Xia Z. Bejjani B. A.. (2009). Genomic and genic deletions of the FOX gene cluster on 16q24.1 and inactivating mutations of FOXF1 cause alveolar capillary dysplasia and other malformations. Am. J. Hum. Genet. 84, 780–791. 10.1016/j.ajhg.2009.05.005 19500772 PMC2694971

[B49] Sullivan D. I. Kass D. J. (2019). Signals and signposts: biomarkers in IPF and PAH at the crossroads of clinical relevance. Respirology 24, 1044–1045. 10.1111/resp.13694 31486582 PMC8491577

[B50] Szklarczyk D. Morris J. H. Cook H. Kuhn M. Wyder S. Simonovic M.. (2017). The STRING database in 2017: quality-controlled protein-protein association networks, made broadly accessible. Nucleic Acids Res. 45, D362–D368. 10.1093/nar/gkw937 27924014 PMC5210637

[B51] Takemoto A. Kimura K. Yokoyama S. Hanaoka F. (2004). Cell cycle-dependent phosphorylation, nuclear localization, and activation of human condensin. J Biol Chem. 279, 4551–4559. 10.1074/jbc.M310925200 14607834

[B52] Tang H. Babicheva A. Mcdermott K. M. Gu Y. Ayon R. J. Song S.. (2018). Endothelial HIF-2alpha contributes to severe pulmonary hypertension due to endothelial-to-mesenchymal transition. Am. J. Physiol. Lung Cell Mol. Physiol. 314, L256–L275. 10.1152/ajplung.00096.2017 29074488 PMC5866501

[B53] Taz T. A. Ahmed K. Paul B. K. Al-Zahrani F. A. Mahmud S. M. H. Moni M. A. (2021). Identification of biomarkers and pathways for the SARS-CoV-2 infections that make complexities in pulmonary arterial hypertension patients. Brief. Bioinform. 22, 1451–1465. 10.1093/bib/bbab026 33611340 PMC7929374

[B54] Van De Veerdonk M. C. Kind T. Marcus J. T. Mauritz G. J. Heymans M. W. Bogaard H. J.. (2011). Progressive right ventricular dysfunction in patients with pulmonary arterial hypertension responding to therapy. J. Am. Coll. Cardiol. 58, 2511–2519. 10.1016/j.jacc.2011.06.068 22133851

[B55] Van De Veerdonk M. C. Marcus J. T. Westerhof N. De Man F. S. Boonstra A. Heymans M. W.. (2015). Signs of right ventricular deterioration in clinically stable patients with pulmonary arterial hypertension. Chest 147, 1063–1071. 10.1378/chest.14-0701 25376008

[B56] Wishart D. S. Feunang Y. D. Guo A. C. Lo E. J. Marcu A. Grant J. R.. (2018). DrugBank 5.0: a major update to the DrugBank database for 2018. Nucleic Acids Res. 46, D1074–D1082. 10.1093/nar/gkx1037 29126136 PMC5753335

[B57] Xia J. Gill E. E. Hancock R. E. (2015). NetworkAnalyst for statistical, visual and network-based meta-analysis of gene expression data. Nat. Protoc. 10, 823–844. 10.1038/nprot.2015.052 25950236

[B58] Xu Z. Ruan J. Pan L. Chen C. (2021). Candidate genes identified in systemic sclerosis-related pulmonary arterial hypertension were associated with immunity, inflammation, and cytokines. Cardiovasc. Ther. 2021:6651009. 10.1155/2021/6651009 33680092 PMC7906811

[B59] Yang L. Liang H. Meng X. Shen L. Guan Z. Hei B.. (2020). mmu_circ_0000790 is involved in pulmonary vascular remodeling in mice with HPH via MicroRNA-374c-Mediated FOXC1. Mol. Ther. Nucleic Acids 20, 292–307. 10.1016/j.omtn.2019.12.027 32199127 PMC7082500

[B60] Yang L. Liang H. Shen L. Guan Z. Meng X. (2019). LncRNA Tug1 involves in the pulmonary vascular remodeling in mice with hypoxic pulmonary hypertension via the microRNA-374c-mediated Foxc1. Life Sci. 237:116769. 10.1016/j.lfs.2019.116769 31422096

[B61] Yang X. Deignan J. L. Qi H. Zhu J. Qian S. Zhong J.. (2009). Validation of candidate causal genes for obesity that affect shared metabolic pathways and networks. Nat Genet. 41, 415–423. 10.1038/ng.325 19270708 PMC2837947

[B62] Zeng Y. Li N. Zheng Z. Chen R. Peng M. Liu W.. (2021). Screening of hub genes associated with pulmonary arterial hypertension by integrated bioinformatic analysis. Biomed. Res. Int. 2021:6626094. 10.1155/2021/6626094 33816621 PMC8010527

[B63] Zhang L. Zeng X. X. Li Y. M. Chen S. K. Tang L. Y. Wang N.. (2021). Keratin 1 attenuates hypoxic pulmonary artery hypertension by suppressing pulmonary artery media smooth muscle expansion. Acta Physiol. 231:e13558. 10.1111/apha.13558 32920982

[B64] Zhang T. Huang C. Luo H. Li J. Huang H. Liu X.. (2021). Identification of key genes and immune profile in limited cutaneous systemic sclerosis-associated pulmonary arterial hypertension by bioinformatics analysis. Life Sci. 271:119151. 10.1016/j.lfs.2021.119151 33539912

[B65] Zhang X. Wang X. Wu J. Peng J. Deng X. Shen Y.. (2018). The diagnostic values of circulating miRNAs for hypertension and bioinformatics analysis. Biosci. Rep. 38:525. 10.1042/BSR20180525 29961674 PMC6147777

[B66] Zhao Y. D. Yun H. Z. H. Peng J. Yin L. Chu L. Wu L.. (2014). De novo synthesize of bile acids in pulmonary arterial hypertension lung. Metabolomics 10, 1169–1175. 10.1007/s11306-014-0653-y 25374487 PMC4213391

[B67] Zhong Y. Catheline D. Houeijeh A. Sharma D. Du L. Besengez C.. (2018). Maternal omega-3 PUFA supplementation prevents hyperoxia-induced pulmonary hypertension in the offspring. Am. J. Physiol. Lung Cell Mol. Physiol. 315, L116–L132. 10.1152/ajplung.00527.2017 29597832

[B68] Zhou Y. Fang X. L. Zhang Y. Feng Y. N. Wang S. S. (2020). miR-20a-5p promotes pulmonary artery smooth muscle cell proliferation and migration by targeting ABCA1. J. Biochem. Mol. Toxicol. 34:e22589. 10.1002/jbt.22589 32720422

